# Cytosinium orotate dihydrate

**DOI:** 10.1107/S1600536812049057

**Published:** 2012-12-05

**Authors:** Gustavo Portalone

**Affiliations:** a Chemistry Department, "Sapienza" University of Rome, P.le A. Moro, 5, I-00185 Rome, Italy

## Abstract

The title compound, C_4_H_6_N_3_O^+^·C_5_H_3_N_2_O_4_^−^·2H_2_O or Cyt^+^·Or^−^·2H_2_O, was synthesized by a reaction between cytosine (4-amino-2-hy­droxy­pyrimidine, Cyt) and orotic acid (2,4-dihy­droxy-6-carb­oxy­pyrimidine, Or) in aqueous solution. The two ions are joined by two N^+^—H⋯O^−^ (±)-(CAHB) hydrogen bonds, forming a dimer with graph-set motif *R*_2_^2^(8). In the crystal, the ion pairs of the asymmetric unit are joined by four N—H⋯O inter­actions to adjacent dimers, forming hydrogen-bonded rings with *R*_2_^2^(8) graph-set motif in a two-dimensional network. The formation of the three-dimensional array is facilitated by water mol­ecules, which act as bridges between structural sub-units linked in *R*_3_^2^(8) and *R*_3_^2^(7) hydrogen-bonded rings. The orotate anion is essentially planar, as the dihedral angle between the planes defined by the carboxylate group and the uracil fragment is 4.0 (4)°.

## Related literature

For the supra­molecular association in proton-transfer adducts containing mol­ecules of biological inter­est, see: Portalone & Colapietro (2007 ▶, 2009 ▶); Portalone (2010 ▶, 2011 ▶); Portalone & Irrera (2011 ▶). For the crystal structure of neutral cytosine, see: McClure & Craven (1973 ▶). For the crystal structures of orotic acid and its salts, see: Lutz (2001 ▶); Portalone (2008 ▶); Solbakk (1971 ▶). For computation of ring patterns formed by hydrogen bonds in crystal structures, see: Bernstein *et al.* (1995 ▶).
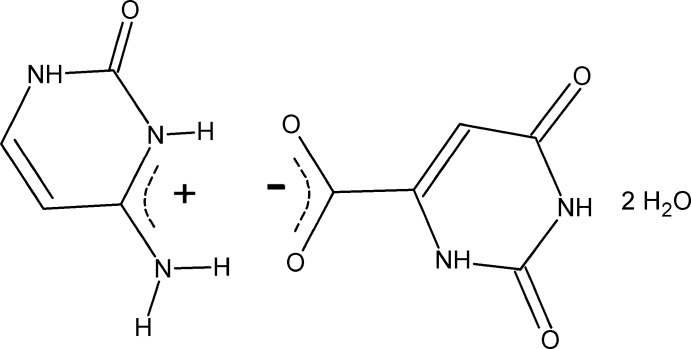


## Experimental

### 

#### Crystal data

C_4_H_6_N_3_O^+^·C_5_H_3_N_2_O_4_^−^·2H_2_O
*M* *_r_* = 303.24
Monoclinic, 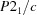

*a* = 5.1486 (2) Å
*b* = 15.1631 (6) Å
*c* = 16.4206 (7) Å
β = 90.562 (3)°
*V* = 1281.87 (9) Å^3^
*Z* = 4
Mo *K*α radiation
μ = 0.14 mm^−1^
*T* = 298 K
0.15 × 0.10 × 0.10 mm

#### Data collection

Oxford Diffraction Xcalibur S CCD diffractometer
Absorption correction: multi-scan (*CrysAlis RED*; Oxford Diffraction, 2006 ▶) *T*_min_ = 0.980, *T*_max_ = 0.987
27833 measured reflections
2328 independent reflections
1954 reflections with *I* > 2σ(*I*)
*R*_int_ = 0.034

#### Refinement

*R*[*F* ^2^ > 2σ(*F* ^2^)] = 0.046
*wR*(*F* ^2^) = 0.125
*S* = 1.13
2328 reflections
228 parameters
4 restraints
H atoms treated by a mixture of independent and constrained refinement
Δρ_max_ = 0.20 e Å^−3^
Δρ_min_ = −0.21 e Å^−3^

### 

Data collection: *CrysAlis CCD* (Oxford Diffraction, 2006 ▶); cell refinement: *CrysAlis RED* (Oxford Diffraction, 2006 ▶); data reduction: *CrysAlis RED*; program(s) used to solve structure: *SIR97* (Altomare *et al.*, 1999 ▶); program(s) used to refine structure: *SHELXL97* (Sheldrick, 2008 ▶); molecular graphics: *WinGX* (Farrugia, 2012 ▶); software used to prepare material for publication: *WinGX* (Farrugia, 2012 ▶).

## Supplementary Material

Crystal structure: contains datablock(s) I, global. DOI: 10.1107/S1600536812049057/rz5024sup1.cif

Structure factors: contains datablock(s) I. DOI: 10.1107/S1600536812049057/rz5024Isup2.hkl

Supplementary material file. DOI: 10.1107/S1600536812049057/rz5024Isup3.cml

Additional supplementary materials:  crystallographic information; 3D view; checkCIF report

## Figures and Tables

**Table 1:** Hydrogen-bond geometry (Å, °)

*D*—H⋯*A*	*D*—H	H⋯*A*	*D*⋯*A*	*D*—H⋯*A*
N7—H7⋯O1^i^	0.76 (2)	2.05 (2)	2.809 (2)	172 (2)
N10—H10*A*⋯O3	0.97 (3)	1.90 (3)	2.871 (2)	173 (2)
N10—H10*B*⋯O6*W*	0.87 (2)	2.05 (2)	2.876 (3)	158 (2)
N9—H9⋯O4	0.86 (3)	1.87 (3)	2.7299 (19)	176 (2)
N1—H1⋯O5^ii^	0.88 (2)	2.20 (2)	3.051 (2)	165.6 (17)
N3—H3⋯O1^iii^	0.93 (2)	1.93 (2)	2.8624 (18)	179.3 (19)
O6*W*—H61⋯O2^ii^	0.85 (2)	1.99 (2)	2.806 (2)	163 (4)
O6*W*—H62⋯O7*W* ^iv^	0.87 (2)	2.07 (3)	2.873 (4)	154 (4)
O7*W*—H71⋯O4	0.90 (2)	1.97 (2)	2.867 (2)	173 (4)
O7*W*—H72⋯O5	0.90 (2)	2.27 (3)	2.979 (2)	136 (3)

Symmetry codes: (i) 
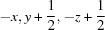
; (ii) 
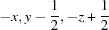
; (iii) 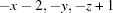; (iv) 
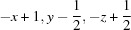
.

**Fig. 1.**
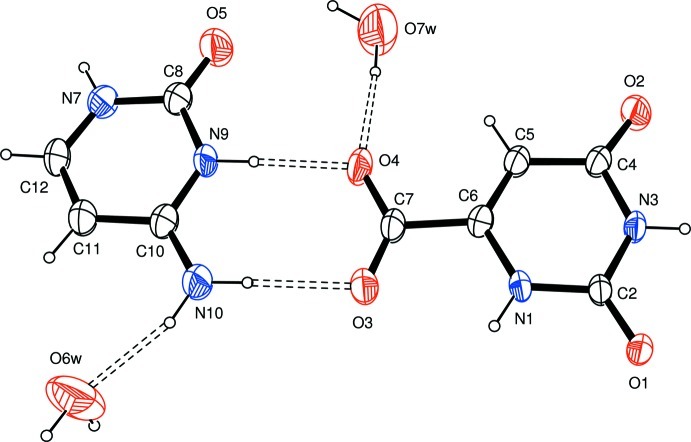
The asymmetric unit of the title compound. Displacements ellipsoids are at the 50% probability level. The asymmetric unit was selected so that the two ions are linked by N+.—H···O- hydrogen bonds. H atoms are shown as small spheres of arbitrary radii. Hydrogen bonds are indicated by dashed lines.

**Fig. 2.**
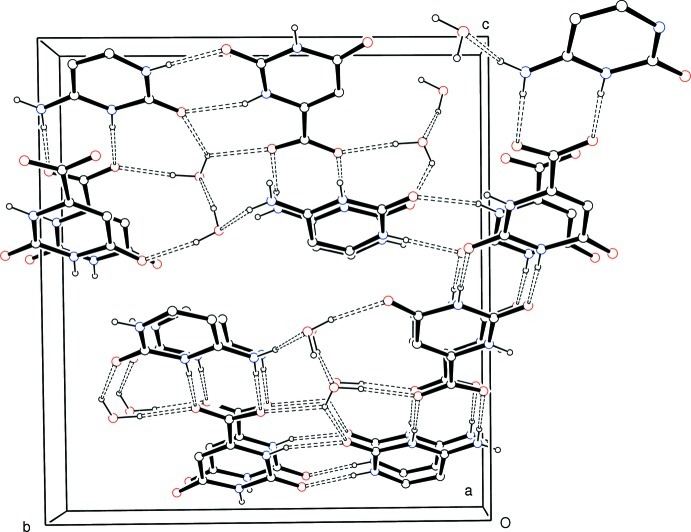
Crystal packing diagram for (I) viewed approximately down a. All atoms are shown as small spheres of arbitrary radii. For the sake of clarity, H atoms not involved in hydrogen bonding have been omitted. Hydrogen bonding is indicated by dashed lines.

## supplementary crystallographic information

### Comment

As part of our ongoing interest in supramolecular architectures of biologically important proton-transfer compounds (Portalone & Colapietro, 2007, 2009; Portalone, 2010, 2011; Portalone & Irrera, 2011), cytosinium orotate dihydrate, C_4_H_6_N_3_O^+^. C_5_H_3_N_2_O_4_^-^.2 H_2_O, Cyt^+^Or^-^, has been synthesized by a reaction between cytosine (4-amino-2-hydroxypyrimidine, Cyt) and orotic acid (2,4-dihydroxy-6-carboxypyrimidine, Or) in water solution.

The title compund crystallizes in the monoclinic space group P21/c, with one protonated aminooxo tautomer (Cyt^+^), one diketo anionic tautomer (Or^-^) and two water molecules of crystallization (Fig 1).

In Cyt^+^ cation protonation occurs at the N9 atom. The molecular geometry is quite similar to those found in previously reported structures of cytosinium salts of organic acids. In particular, as shown in previous studies (Portalone & Colapietro, 2009; Portalone, 2011), the internal angle C8-N9-C10 is sensitive to protonation, being larger (124.86 (15)°) than the corresponding one in the neutral cytosine molecule (119.4 (2)°, McClure & Craven, 1973). The cytosine ring is slightly puckered with N7, C8 and C10 atoms deviating from the mean plane of the other atoms by 0.028 (1), -0.019 (1) and 0.023 (1) Å, respectively. Nevertheless, the variation observed for the C10—N9 and C10—N10 bond distances in passing from the protonated (1.351 (2), 1.302 (2) Å) to the neutral cytosine molecule (1.341 (2), 1.326 (2) Å, McClure & Craven, 1973) suggests some degree of delocalization of π-electron density through the amidinium moiety.

In Or^-^ anion the pyrimidine ring is essentially planar and the carboxylate group forms dihedral angle of 4.0 (4)° with the mean plane of the uracil fragment, which is close to the value observed in orotic acid (2.2 (2)°, Portalone, 2008). In this anion bond lengths and bond angles of the heteroaromatic ring are in accord with those obtained for other similar structures of ammonium orotate monohydrate (Solbakk, 1971), lithium orotate monohydrate (Lutz, 2001) and benzamidinium orotate hemihydrate (Portalone, 2010). Bond distances around atom C7 indicate a carboxylate group with delocalization of the negative charge between atoms O3 and O4.

The two ions are joined by two N^+^—H···O^-^ (±)CAHB hydrogen bonds to form a dimer with graph-set motif R^2^_2_(8) (Bernstein *et al.*, 1995). In the crystal, the ion pairs of the asymmetric unit are joined by four N—H···O interactions with D···A distances ranging from 2.809 (2) to 3.051 (2) Å. Adjacent ion pairs form hydrogen-bonded rings with R^2^_2_(8) graph-set motif in a bi-dimensional network (Table 2). The formation of the three-dimensional array is facilitated by water molecules, which act as bridges between structural subunits linked in R^2^_3_(8) and R^2^_3_(7) hydrogen-bonded rings. Water molecules play an important role in the cohesion and the stability of the crystal structure: they are involved in four O—H···O hydrogen bonds, three connecting two Or^-^ anions and one Cyt^+^ cation as donor (O6W—H61···O2, O7W—H71···O4 and O7W—H72···O5), and one between two water molecules (O6W—H62···O7W).

### Experimental

A water solution (6 ml) of cytosine (0.01 mmol, Fluka at 96% purity) was mixed with an aqueous solution (5 ml) containing orotic acid (0.01 mmol, Sigma Aldrich, 98%), and the resulting mixture was heated under reflux with stirring for 3 h. After cooling the solution to an ambient temperature, colourless crystals suitable for single-crystal X-ray diffraction were grown by slow evaporation of the solvent after two weeks.

### Refinement

All H atoms were identified in difference Fourier maps, but for refinement all C-bound H atoms were placed in calculated positions, with C—H = 0.93 Å and refined as riding on their carrier atoms. The *U*_iso_ values were kept equal to 1.2*U*_eq_(C). Positional and thermal parameters of H atoms attached to N atoms were refined freely, giving N—H distances in the range 0.76 (2) - 0.97 (3) Å. For the water molecules, the O—H distances of the H atoms attached to O6W and O7W were restrained to 0.85 (2) - 0.90 (2) Å, and the H atoms were refined with *U*_iso_ equal to 1.5*U*_eq_(O).

### Crystal data

**Table:**

C_4_H_6_N_3_O^+^·C_5_H_3_N_2_O_4_^−^·2H_2_O	*F*(000) = 632
*M**_r_* = 303.24	*D*_x_ = 1.571 Mg m^−^^3^
Monoclinic, *P*2_1_/*c*	Mo *K*α radiation, λ = 0.71069 Å
Hall symbol: -P 2ybc	Cell parameters from 14285 reflections
*a* = 5.1486 (2) Å	θ = 2.7–29.0°
*b* = 15.1631 (6) Å	µ = 0.14 mm^−^^1^
*c* = 16.4206 (7) Å	*T* = 298 K
β = 90.562 (3)°	Tablets, colourless
*V* = 1281.87 (9) Å^3^	0.15 × 0.10 × 0.10 mm
*Z* = 4	

### Data collection

**Table:**

Oxford Diffraction Xcalibur S CCD diffractometer	2328 independent reflections
Radiation source: Enhance (Mo) X-ray source	1954 reflections with *I* > 2σ(*I*)
Graphite monochromator	*R*_int_ = 0.034
Detector resolution: 16.0696 pixels mm^-1^	θ_max_ = 25.3°, θ_min_ = 2.7°
ω and φ scans	*h* = −6→6
Absorption correction: multi-scan (*CrysAlis RED*; Oxford Diffraction, 2006)	*k* = −18→18
*T*_min_ = 0.980, *T*_max_ = 0.987	*l* = −19→19
27833 measured reflections	

### Refinement

**Table:**

Refinement on *F*^2^	Primary atom site location: structure-invariant direct methods
Least-squares matrix: full	Secondary atom site location: difference Fourier map
*R*[*F*^2^ > 2σ(*F*^2^)] = 0.046	Hydrogen site location: inferred from neighbouring sites
*wR*(*F*^2^) = 0.125	H atoms treated by a mixture of independent and constrained refinement
*S* = 1.13	*w* = 1/[σ^2^(*F*_o_^2^) + (0.0662*P*)^2^ + 0.3271*P*] where *P* = (*F*_o_^2^ + 2*F*_c_^2^)/3
2328 reflections	(Δ/σ)_max_ < 0.001
228 parameters	Δρ_max_ = 0.20 e Å^−^^3^
4 restraints	Δρ_min_ = −0.21 e Å^−^^3^

### Special details

**Table:**

Geometry. All s.u.'s (except the s.u. in the dihedral angle between two l.s. planes) are estimated using the full covariance matrix. The cell s.u.'s are taken into account individually in the estimation of s.u.'s in distances, angles and torsion angles; correlations between s.u.'s in cell parameters are only used when they are defined by crystal symmetry. An approximate (isotropic) treatment of cell s.u.'s is used for estimating s.u.'s involving l.s. planes.
Refinement. Refinement of F^2^ against ALL reflections. The weighted R-factor wR and goodness of fit S are based on F^2^, conventional R-factors R are based on F, with F set to zero for negative F^2^. The threshold expression of F^2^ > 2σ(F^2^) is used only for calculating R-factors(gt) etc. and is not relevant to the choice of reflections for refinement. R-factors based on F^2^ are statistically about twice as large as those based on F, and R- factors based on ALL data will be even larger.

### Fractional atomic coordinates and isotropic or equivalent isotropic displacement parameters (Å^2^)

**Table:**

	*x*	*y*	*z*	*U*_iso_*/*U*_eq_	
O5	0.2952 (3)	0.32438 (9)	0.16089 (11)	0.0590 (5)	
N7	0.6235 (3)	0.26032 (12)	0.09082 (11)	0.0419 (5)	
H7	0.678 (5)	0.3059 (15)	0.0814 (14)	0.046 (6)*	
C8	0.4062 (4)	0.25827 (12)	0.13766 (12)	0.0382 (5)	
N10	0.3426 (4)	0.02351 (12)	0.15490 (11)	0.0453 (5)	
H10A	0.193 (5)	0.0195 (17)	0.1906 (16)	0.067 (7)*	
H10B	0.427 (4)	−0.0231 (16)	0.1400 (13)	0.051 (6)*	
N9	0.3208 (3)	0.17444 (10)	0.15622 (10)	0.0349 (4)	
H9	0.188 (5)	0.1701 (14)	0.1872 (16)	0.055 (7)*	
C10	0.4331 (3)	0.09908 (11)	0.13046 (11)	0.0331 (4)	
C11	0.6503 (4)	0.10687 (13)	0.07734 (12)	0.0391 (5)	
H11	0.7293	0.0571	0.0555	0.050 (6)*	
C12	0.7361 (4)	0.18745 (13)	0.06001 (12)	0.0398 (5)	
H12	0.8776	0.1937	0.0257	0.048 (6)*	
O1	−0.7741 (2)	−0.06618 (8)	0.44582 (8)	0.0403 (4)	
O2	−0.8140 (3)	0.23043 (8)	0.45689 (9)	0.0485 (4)	
O3	−0.0719 (3)	0.01695 (8)	0.27044 (9)	0.0474 (4)	
O4	−0.0853 (3)	0.16324 (8)	0.26139 (8)	0.0440 (4)	
N1	−0.4837 (3)	0.01212 (10)	0.36986 (9)	0.0322 (4)	
H1	−0.419 (4)	−0.0379 (14)	0.3528 (11)	0.039 (5)*	
C2	−0.6871 (3)	0.00513 (11)	0.42196 (10)	0.0293 (4)	
N3	−0.7902 (3)	0.08258 (9)	0.44828 (9)	0.0318 (4)	
H3	−0.932 (4)	0.0768 (13)	0.4826 (13)	0.042 (5)*	
C4	−0.7074 (4)	0.16627 (11)	0.42733 (11)	0.0323 (4)	
C5	−0.4941 (4)	0.16797 (11)	0.37152 (11)	0.0325 (4)	
H5	−0.4283	0.2217	0.3539	0.039*	
C6	−0.3900 (3)	0.09262 (11)	0.34486 (10)	0.0289 (4)	
C7	−0.1609 (3)	0.09014 (11)	0.28677 (10)	0.0307 (4)	
O6W	0.7353 (4)	−0.09802 (12)	0.10206 (16)	0.0836 (7)	
H61	0.733 (8)	−0.1472 (17)	0.078 (2)	0.125*	
H62	0.812 (8)	−0.112 (3)	0.1479 (16)	0.125*	
O7W	−0.1266 (4)	0.35051 (13)	0.28142 (14)	0.0868 (7)	
H71	−0.125 (8)	0.2911 (13)	0.278 (2)	0.130*	
H72	−0.033 (7)	0.373 (2)	0.2403 (18)	0.130*	

### Atomic displacement parameters (Å^2^)

**Table:**

	*U*^11^	*U*^22^	*U*^33^	*U*^12^	*U*^13^	*U*^23^
O5	0.0601 (10)	0.0344 (8)	0.0831 (12)	0.0029 (6)	0.0346 (9)	0.0003 (7)
N7	0.0389 (10)	0.0309 (9)	0.0561 (11)	−0.0062 (7)	0.0192 (8)	0.0093 (8)
C8	0.0359 (11)	0.0339 (10)	0.0448 (11)	−0.0012 (8)	0.0129 (8)	0.0059 (8)
N10	0.0468 (11)	0.0310 (9)	0.0585 (11)	−0.0032 (8)	0.0238 (9)	0.0019 (8)
N9	0.0295 (9)	0.0344 (9)	0.0411 (9)	−0.0020 (6)	0.0149 (7)	0.0045 (6)
C10	0.0297 (10)	0.0338 (9)	0.0359 (10)	−0.0031 (7)	0.0069 (8)	0.0020 (7)
C11	0.0368 (11)	0.0377 (10)	0.0431 (11)	0.0007 (8)	0.0161 (8)	0.0020 (8)
C12	0.0323 (11)	0.0445 (11)	0.0431 (11)	−0.0010 (8)	0.0167 (8)	0.0082 (9)
O1	0.0435 (8)	0.0247 (6)	0.0534 (8)	0.0003 (5)	0.0287 (6)	0.0030 (6)
O2	0.0573 (9)	0.0240 (6)	0.0648 (9)	0.0027 (6)	0.0322 (7)	−0.0025 (6)
O3	0.0467 (9)	0.0344 (7)	0.0616 (9)	0.0021 (6)	0.0304 (7)	0.0024 (6)
O4	0.0451 (8)	0.0338 (7)	0.0536 (8)	−0.0068 (6)	0.0281 (7)	0.0031 (6)
N1	0.0325 (9)	0.0256 (8)	0.0388 (9)	0.0000 (6)	0.0175 (7)	−0.0004 (6)
C2	0.0282 (10)	0.0266 (9)	0.0332 (9)	−0.0003 (7)	0.0130 (7)	0.0020 (7)
N3	0.0306 (8)	0.0274 (8)	0.0376 (8)	0.0002 (6)	0.0184 (7)	0.0009 (6)
C4	0.0343 (10)	0.0246 (8)	0.0383 (10)	−0.0016 (7)	0.0118 (8)	0.0018 (7)
C5	0.0339 (10)	0.0253 (8)	0.0385 (10)	−0.0040 (7)	0.0134 (8)	0.0028 (7)
C6	0.0260 (9)	0.0312 (9)	0.0297 (9)	−0.0043 (7)	0.0067 (7)	0.0044 (7)
C7	0.0268 (9)	0.0320 (9)	0.0334 (9)	−0.0030 (7)	0.0095 (7)	0.0014 (7)
O6W	0.0904 (15)	0.0403 (9)	0.1201 (19)	0.0107 (9)	0.0027 (13)	−0.0186 (11)
O7W	0.0985 (15)	0.0480 (10)	0.1149 (17)	−0.0042 (10)	0.0569 (13)	−0.0065 (11)

### Geometric parameters (Å, º)

**Table:**

O5—C8	1.217 (2)	O3—C7	1.231 (2)
N7—C12	1.349 (2)	O4—C7	1.248 (2)
N7—C8	1.364 (2)	N1—C2	1.363 (2)
N7—H7	0.76 (2)	N1—C6	1.377 (2)
C8—N9	1.380 (2)	N1—H1	0.88 (2)
N10—C10	1.302 (2)	C2—N3	1.361 (2)
N10—H10A	0.97 (3)	N3—C4	1.383 (2)
N10—H10B	0.87 (2)	N3—H3	0.93 (2)
N9—C10	1.351 (2)	C4—C5	1.437 (2)
N9—H9	0.86 (3)	C5—C6	1.338 (2)
C10—C11	1.430 (2)	C5—H5	0.9300
C11—C12	1.331 (3)	C6—C7	1.525 (2)
C11—H11	0.9300	O6W—H61	0.845 (19)
C12—H12	0.9300	O6W—H62	0.871 (19)
O1—C2	1.2357 (19)	O7W—H71	0.902 (19)
O2—C4	1.220 (2)	O7W—H72	0.898 (19)
			
C12—N7—C8	123.41 (17)	C2—N1—H1	115.5 (13)
C12—N7—H7	120.5 (18)	C6—N1—H1	122.5 (13)
C8—N7—H7	116.1 (18)	O1—C2—N3	120.71 (14)
O5—C8—N7	123.23 (17)	O1—C2—N1	123.39 (15)
O5—C8—N9	122.53 (16)	N3—C2—N1	115.89 (14)
N7—C8—N9	114.23 (16)	C2—N3—C4	126.19 (14)
C10—N10—H10A	121.9 (15)	C2—N3—H3	114.9 (12)
C10—N10—H10B	116.6 (15)	C4—N3—H3	118.9 (12)
H10A—N10—H10B	121 (2)	O2—C4—N3	119.45 (15)
C10—N9—C8	124.86 (15)	O2—C4—C5	126.07 (15)
C10—N9—H9	117.9 (15)	N3—C4—C5	114.48 (14)
C8—N9—H9	117.3 (15)	C6—C5—C4	120.29 (15)
N10—C10—N9	119.49 (16)	C6—C5—H5	119.9
N10—C10—C11	123.05 (17)	C4—C5—H5	119.9
N9—C10—C11	117.46 (15)	C5—C6—N1	121.14 (15)
C12—C11—C10	117.99 (17)	C5—C6—C7	122.74 (15)
C12—C11—H11	121.0	N1—C6—C7	116.11 (15)
C10—C11—H11	121.0	O3—C7—O4	127.60 (15)
C11—C12—N7	121.83 (16)	O3—C7—C6	116.76 (15)
C11—C12—H12	119.1	O4—C7—C6	115.65 (15)
N7—C12—H12	119.1	H61—O6W—H62	102 (3)
C2—N1—C6	121.99 (15)	H71—O7W—H72	109 (3)
			
C12—N7—C8—O5	−175.0 (2)	N1—C2—N3—C4	−0.2 (3)
C12—N7—C8—N9	4.6 (3)	C2—N3—C4—O2	−178.38 (18)
O5—C8—N9—C10	178.4 (2)	C2—N3—C4—C5	0.9 (3)
N7—C8—N9—C10	−1.2 (3)	O2—C4—C5—C6	178.4 (2)
C8—N9—C10—N10	176.86 (19)	N3—C4—C5—C6	−0.8 (3)
C8—N9—C10—C11	−2.7 (3)	C4—C5—C6—N1	0.1 (3)
N10—C10—C11—C12	−176.2 (2)	C4—C5—C6—C7	−178.95 (16)
N9—C10—C11—C12	3.4 (3)	C2—N1—C6—C5	0.7 (3)
C10—C11—C12—N7	−0.2 (3)	C2—N1—C6—C7	179.75 (16)
C8—N7—C12—C11	−4.0 (3)	C5—C6—C7—O3	175.70 (18)
C6—N1—C2—O1	−179.52 (17)	N1—C6—C7—O3	−3.4 (2)
C6—N1—C2—N3	−0.6 (3)	C5—C6—C7—O4	−4.2 (3)
O1—C2—N3—C4	178.74 (18)	N1—C6—C7—O4	176.72 (16)

### Hydrogen-bond geometry (Å, º)

**Table:**

*D*—H···*A*	*D*—H	H···*A*	*D*···*A*	*D*—H···*A*
N7—H7···O1^i^	0.76 (2)	2.05 (2)	2.809 (2)	172 (2)
N10—H10*A*···O3	0.97 (3)	1.90 (3)	2.871 (2)	173 (2)
N10—H10*B*···O6*W*	0.87 (2)	2.05 (2)	2.876 (3)	158 (2)
N9—H9···O4	0.86 (3)	1.87 (3)	2.7299 (19)	176 (2)
N1—H1···O5^ii^	0.88 (2)	2.20 (2)	3.051 (2)	165.6 (17)
N3—H3···O1^iii^	0.93 (2)	1.93 (2)	2.8624 (18)	179.3 (19)
O6*W*—H61···O2^ii^	0.85 (2)	1.99 (2)	2.806 (2)	163 (4)
O6*W*—H62···O7*W*^iv^	0.87 (2)	2.07 (3)	2.873 (4)	154 (4)
O7*W*—H71···O4	0.90 (2)	1.97 (2)	2.867 (2)	173 (4)
O7*W*—H72···O5	0.90 (2)	2.27 (3)	2.979 (2)	136 (3)

Symmetry codes: (i) −*x*, *y*+1/2, −*z*+1/2; (ii) −*x*, *y*−1/2, −*z*+1/2; (iii) −*x*−2, −*y*, −*z*+1; (iv) −*x*+1, *y*−1/2, −*z*+1/2.
